# Low Dose Cyclophosphamide Modulates Tumor Microenvironment by TGF-β Signaling Pathway

**DOI:** 10.3390/ijms21030957

**Published:** 2020-01-31

**Authors:** Hui Zhong, Yifan Lai, Rui Zhang, Abdelkader Daoud, Qingyuan Feng, Jia Zhou, Jing Shang

**Affiliations:** 1 State Key Laboratory of Natural Medicines, China Pharmaceutical University, Nanjing 210009, China; cpuzhonghui@126.com (H.Z.); Yifan_Lai@outlook.com (Y.L.); zhangrui19950825@163.com (R.Z.); 15651715868@163.com (Q.F.); 2 School of Traditional Chinese Pharmacy, China Pharmaceutical University, Nanjing 211198, China; ADCPU99@163.com

**Keywords:** cyclophosphamide, tumor microenvironment, metronomic schedule, TGF-β

## Abstract

The tumor microenvironment has been recently recognized as a critical contributor to cancer progression and anticancer therapy-resistance. Cyclophosphamide (CTX) is a cytotoxic agent commonly used in clinics for the treatment of cancer. Previous reports demonstrated that CTX given at low continuous doses, known as metronomic schedule, mainly targets endothelial cells and circulating Tregs with unknown mechanisms. Here, we investigated the antitumor activity of two different metronomic schedules of CTX along with their corresponding MTD regimen and further explored their effect on immune function and tumor microenvironment. Toxicity evaluation was monitored by overall survival rate, weight loss, and histopathological analysis. A nude mouse model of Lewis lung cancer was established to assess the anti-metastatic effects of CTX in vivo. CD4^+^, CD8^+^, and CD4^+^CD25^+^FoxP3 T cells were selected by flow cytometry analysis. Low and continuous administration of CTX was able to restore immune function via increase of CD4^+^/CD8^+^ T cells and depletion of T regulatory cells, not only in circulatory and splenic compartments, but also at the tumor site. Low-dose CTX also reduced myofibroblasts, accompanied with an increased level of E-cadherin and low N-cadherin, both in the primary tumor and lung through the TGF-β pathway by the downregulated expression of TGF-β receptor 2. Our data may indicate that several other molecular mechanisms of CTX for tumor may be involved in metronomic chemotherapy, besides targeting angiogenesis and regulatory T cells.

## 1. Introduction

In both sexes combined, lung cancer is the most commonly diagnosed cancer (11.6% of the total cases) and the leading cause of cancer death (18.4% of total cancer deaths) in 2018 [1]. Although many current therapies have shown promising results in clinics, only few showed long-term activity because tumor cells rapidly develop “drug resistance” [2]. The main reason for this phenomenon is because tumor exists in an intimate relationship with its surrounding microenvironment, which, in addition to initiating and supporting the tumorigenic process, can also affect the sensitivity of tumor cells to drug treatment. A tumor microenvironment (TME) is composed of cancer cells in association with a variety of other cells—stromal cells, fibroblasts, myofibroblasts, endothelial cells, immunosuppressive leukocytes like regulatory T cells (Tregs) and other suppressor cells, which inhibit immunity through cell contact or secretion of various cytokines [3] (such as transforming growth factor beta (TGF-β) [4]). These cytokines promote tumor cell growth and escape immune attacks by inducing different immunosuppressive cells, such as regulatory T cells (Tregs) and myofibroblasts, which hamper the anti-tumor activity of effector lymphocytes [5]. In general, tumor-associated fibroblasts (TAFs), which include fibroblasts but mostly activated fibroblasts (myofibroblasts) are formed via either fibroblast-myofibroblast transition (FMT) [6] or a transdifferentiation by epithelial-mesenchymal transition (EMT) [7]. These two phenomena are under control of the TGF-β signaling pathway.

Cyclophosphamide (CTX) is an alkylating agent commonly used at high doses in clinics for the treatment of cancer [8]. Conventional CTX chemotherapy, which is generally based on the concept of the maximum tolerated dose (MTD), has recently changed with a low dose-based therapy, in order to not only reduce toxicity but also target tumor vascular endothelial cells leading to tumor regression [9,10]. This chemotherapy schedule, which was first defined as “Metronomic Chemotherapy” (Met) by Douglas Hanahan [11], was proposed to mainly target endothelial cells [12] and circulating Tregs [13,14] with unknown mechanisms. Yet, treatment with cyclophosphamide of tumor-bearing animals resulted in a decreased production of TGF-β [8,15] and anti-metastatic activity. Given the broad range of immunosuppressive activities in TGF-β pathway in the tumor microenvironment, any modulation may have multiple effects. Therefore, we based our investigation on the hypothesis that metronomic CTX may modulate the tumor microenvironment, assuming that it affects TGF-β.

Several models of metronomic CTX schedule have been previously proposed and explored in preclinical settings. Here, an MTD regimen and two different metronomic schedules, Met-1 and Met-2 were adopted. The objective of this study was to investigate the antitumor activity of these schedules and explore the effect of a low-dose regimen on immune function and tumor microenvironment.

## 2. Results

### 2.1. Toxicity Evaluation of Different Regimens

**Figure 1:**
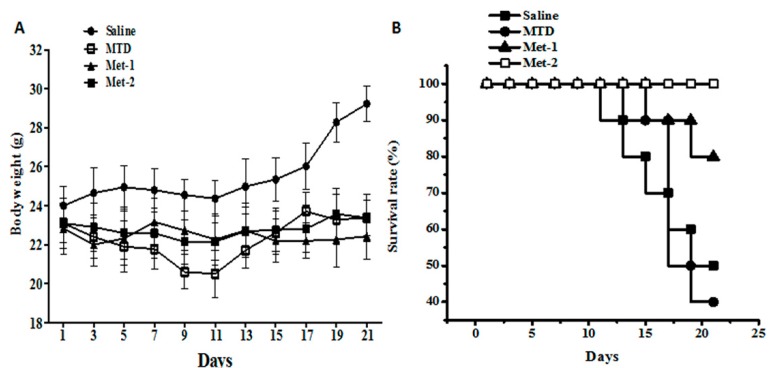
Toxicity evaluation of cyclophosphamide in the LLC model. (**A**) The body weight of mice from each group at the end of the observation period; (**B**) The survival rate of mice from each group at the end of the observation period. C57BL mice were injected with 10^6^ cells in the right flank; after four days, cyclophosphamide at different schedules was given for three weeks. Body weight was measured every other day using a weight scale; the mice were killed on day 22. MTD: 3 doses of 150 mg/kg each, for the first week only; Met-1: 170 mg/kg every 6 days; Met-2: 25 mg/kg every other day.

The three schedules’ toxicity indexes, such as overall survival rate and weight loss, were permanently monitored during the study. Figure 1A shows the body weight assessment done during the study. As demonstrated in this figure, tumor-bearing control mice (saline) had the biggest body weights among all groups. Mice treated with high dose of CTX (MTD) showed decreased body weights compared to the cancer group, especially from day 7 to day 11, which might be due to the toxic effect of the drug. On the contrary, both metronomic schedules (Met-1 and Met-2) had stable weights with slight changes throughout the experiment. The metronomic regimen Met-2 had higher body weights in the end, compared to the Met-1 schedule and that was essentially due to the tumor growth.

Further, overall survival was determined using the Kaplan–Meier curve (Figure 1B). On one hand, the MTD regimen decreased animal survival, compared to the saline group. On the other hand, both metronomic schedules were found to significantly enhance animal survival, compared to both saline and MTD groups. Overall, metronomic schedule 2 (Met-2) had the highest survival rate during the study.

**Figure 2:**
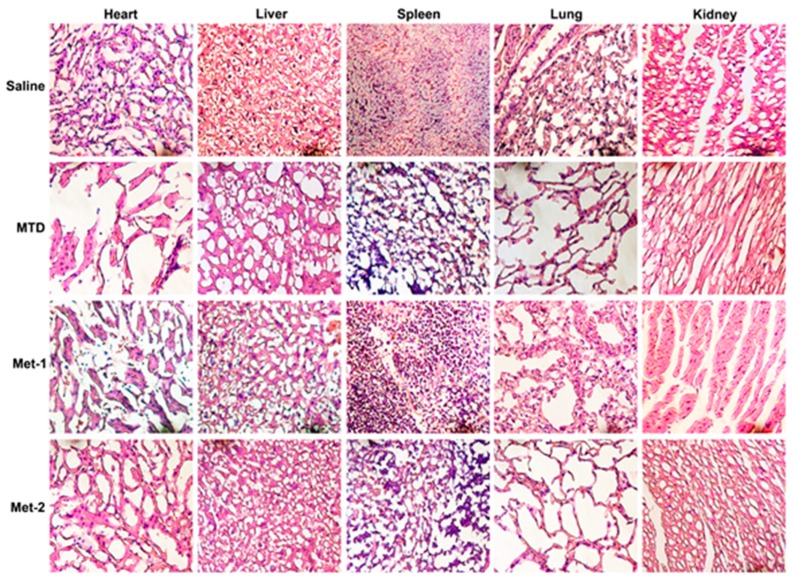
After 21 days of treatment, the major organs (heart, kidney, liver, lung and spleen) were harvested from mice and then stained with H&E. Cyclophosphamide at different schedules was given for three weeks. The mice were killed on day 22 and tissues were collected and H&E staining was performed. MTD: 3 doses of 150 mg/kg each, for the first week only; Met-1: 170 mg/kg every 6 days; Met-2: 25 mg/kg every other day. ×400.

Additionally, to further evaluate the toxic effect of different CTX schedules, tissues such as heart, liver, lung, kidney, and spleen were fixed in 10% neutral buffered paraformaldehyde solution and embedded in paraffin. Sections of 3–5 μm were stained with hematoxylin and eosin (H&E); microscopic evaluation and examination was done by a pathologist (Figure 2).

As revealed in Figure 2, a high-dose CTX (MTD) caused significant cytotoxicity, reflected by the destruction of the architecture of different tissues, especially in the heart, kidney, lung, and spleen. Met-1 showed less toxicity, which was mainly localized in the heart. Met-2 had no detectable toxic effect.

### 2.2. The Antitumor Effect of Different Schedules

**Figure 3:**
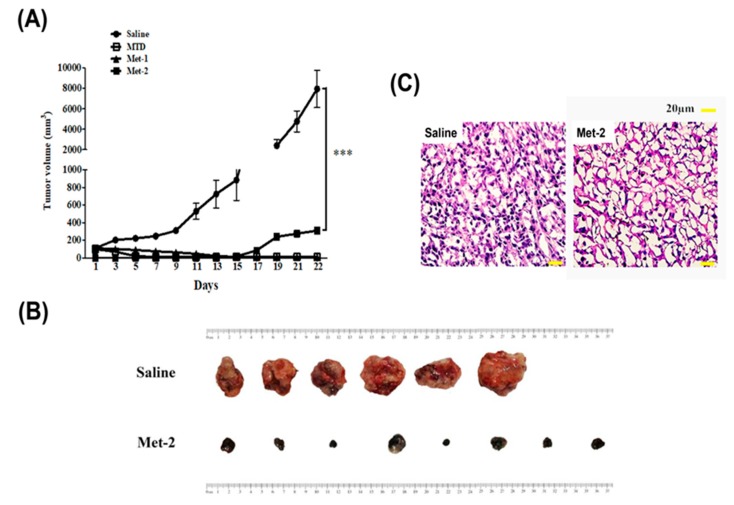
In vivo antitumor activity of cyclophosphamide in mice (C57BL mice) bearing LLC xenograft model. (**A**) Tumor volume of the mice in each group during the observation period. (**B**) After being administered with cyclophosphamide at a dose of 25 mg/kg every other day for 21 days, the mice were sacrificed and the tumors weighed. (**C**) H&E staining of tumor tissue from saline and Met-2 group. ***p < 0.001.

To evaluate the in vivo antitumor efficacy of different schedules, tumor volume in each group was monitored every other day (Figure 3A). Both the MTD and Met-1 schedules caused a significant reduction in tumors, which was undetectable by the end of the study. The Met-2 schedule significant reduced tumor growth throughout the investigation (*p* < 0.001).

In addition, after all animals were sacrificed on day 22, tumors were collected and pictures taken (Figure 3B). As mentioned before, while both MTD and Met-1 treated mice had no tumors by the end of our study, the group treated with Met-2 schedule had tumors that were significantly smaller than those of the saline group. To further confirm the anti-cancer activity of the Met-2 regimen, histopathology analysis using H&E staining was performed on the tumors. Figure 3C confirmed the potential anti-cancer effect of Met-2, compared to the saline group.

### 2.3. The Immunomodulatory Effect of CTX Regimens

**Figure 4:**
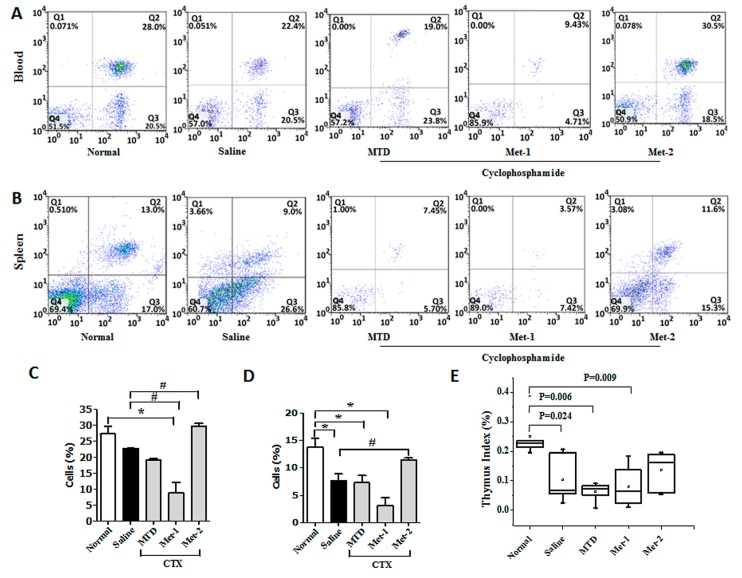
CD3^+^/CD4^+^ T cells in blood (**A**,**C**) and spleen (**B**,**D**) from each group at the end of the observation period were measured by flow cytometry analysis. Fresh blood was collected in EDTA tubes and flow cytometry analysis was performed as described previously. (**E**) Thymus Index. Thymuses were collected and Thymus index was calculated. “*” versus normal group and “#” versus saline group. *p < 0.05, #*p* < 0.05.

It is well documented that a decrease in CD4^+^ and CD8^+^ populations occurred in the peripheral blood of patients suffering from different cancers [16]. Similar observation was also obtained from animal studies [17]. Here, we performed flow cytometry to analyze the level of CD4^+^, CD8^+^ and regulatory T cells (Tregs) in both the blood and the spleen. Figure 4 showed that the CD4^+^ population from blood and spleen was diminished in LLC-bearing mice, in comparison to that of normal counterparts. This depletion may be explained by the decrease of thymic mass (thymus index), as depicted in Figure 4E. The level of CD4^+^ from CTX-treated mice was also ealuated. Previous studies indicated that a high dose of CTX is immunosuppressive and causes profound depletion of T cells due to its toxicity [18]. Our data showed a remarkable reduction of circulatory and splenic CD4^+^ cells, along with a significant decrease of the thymus index. Contrary to the MTD regimen, previous reports have revealed that low-dose CTX can stimulate the immune system in tumor-bearing mice [19]. Figure 4 demonstrates that Met-1 was more potent that MTD is reducing the level of CD4^+^ T cells in both the circulating and splenic compartments. This may reflect the toxic effect of Met-1, which was also observed from the low thymus index (Figure 4E). In contrast to MTD and Met-1 schedules, Met-2 significantly enhanced the number of this population in both blood and spleen with no notable thymus index change in comparison to the normal group (Figure 4E).

**Figure 5:**
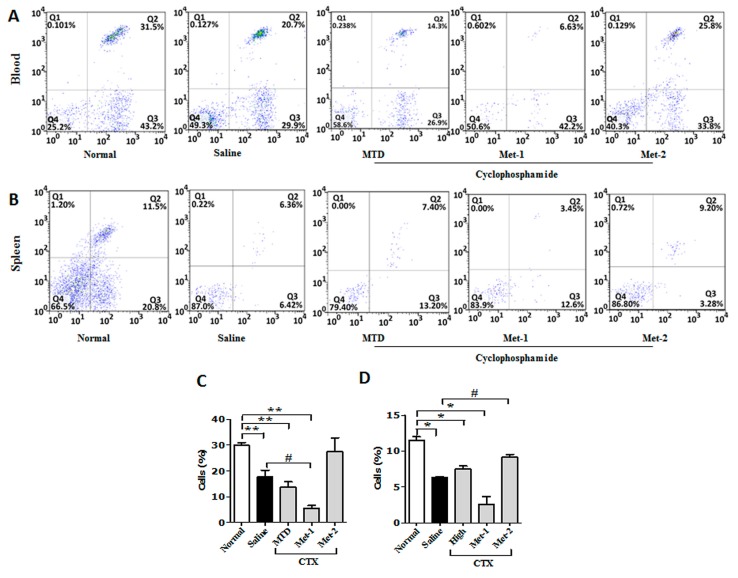
CD3^+^/CD8^+^ T cells in blood (**A**,**C**) and spleen (**B**,**D**) from each group at the end of the observation period were measured by flow cytometry analysis. Fresh blood was collected in EDTA tubes and flow cytometry analysis was performed as described previously. “*” versus normal group and “#” versus saline group. *p < 0.05, #*p* < 0.05, **p < 0.01.

Similarly, less CD8^+^ T cells were detected in tumor-bearing mice, compared to their normal counterparts (Figure 5). High-dose CTX significantly reduced the proportion of CD8^+^ cells in both compartments, especially in the blood. The decrease of this population in the Met-1 group was even greater, confirming non-selective immune suppression by this schedule due to its toxicity. Meanwhile, the Met-2 schedule increased the level of circulating CD8^+^ T cells, but more importantly the splenic ones, in comparison to the saline group (Figure 5).

**Figure 6:**
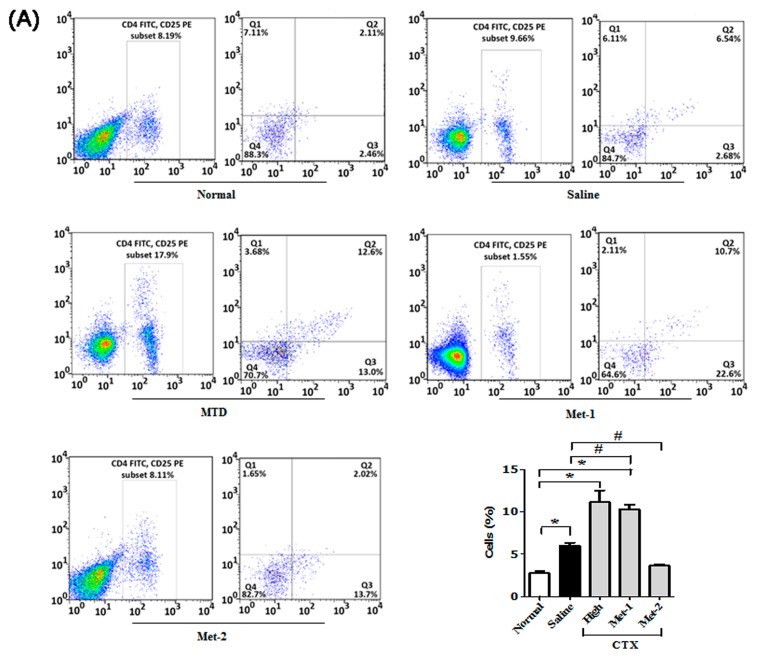

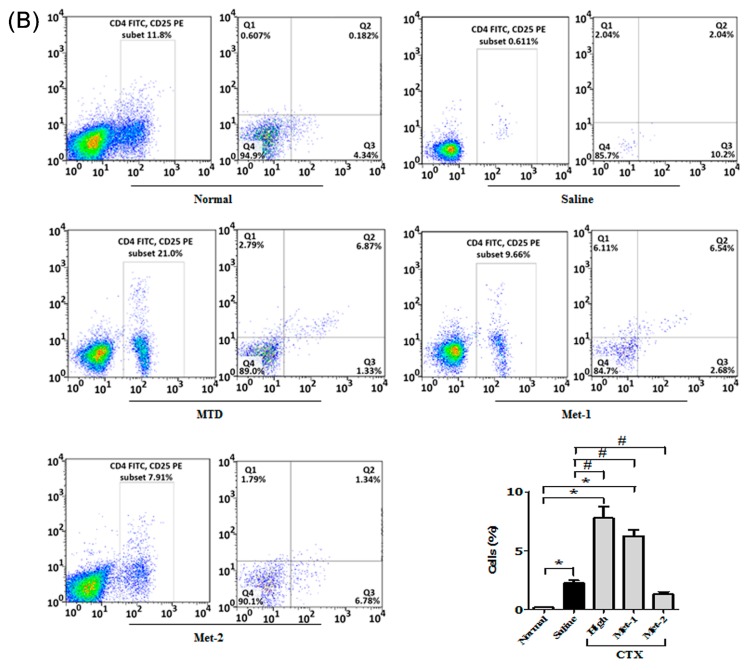
CD4^+^/CD25^+^FoxP3 cells in blood (**A**) and spleen (**B**) from each group at the end of the observation period were measured by flow cytometry analysis. Fresh blood was collected in EDTA tubes and flow cytometry analysis was performed as described previously. “*” versus normal group and “#” versus saline group. *p < 0.05, #*p* < 0.05.

It is becoming increasingly clear that regulatory T cells (Tregs) play a significant role in suppressing tumor-specific immunity [20]. Our data also showed a high frequency of Tregs in both blood and spleen of mice harboring LLC cells (Figure 6A,B).

Many reports have already demonstrated that low-dose CTX can boost the host immune system and restore CD4^+^/CD8^+^ responses via targeting Tregs [21,22,23]. Figure 6 shows that both high dose schedule (MTD) and Met-1 dramatically increased the number of circulatory and splenic Tregs in mice, compared to tumor-free and tumor-bearing control animals. On the contrary, only low and continuous administration of CTX (Met-2) was able to significantly reduce the number of these immunosuppressive cells.

**Figure 7:**
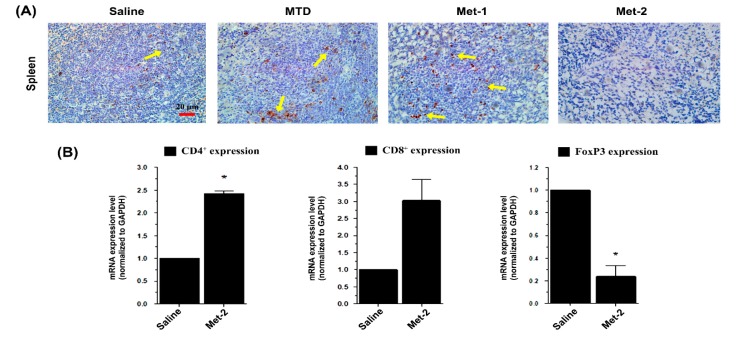
Immunomodulatory effect of cyclophosphamide. (**A**) Immunohistochemistry for FoxP3 marker of Tregs (showed by yellow arrow) in the spleen of tumor-bearing mice, scale bar = 20 μm. (**B**) qPCR analysis for the mRNA levels of CD4, CD8, and FoxP3 in the tumor microenvironment. Spleens were collected and immunohistochemistry performed following the protocol described above. Tumors were collected and total RNA was isolated; then, qPCR was performed as previously described. Met-2, 25 mg/kg every other day. * *p* < 0.05.

In order to confirm the effect of Met-2 regimen on splenic Treg population, we performed immunohistochemistry analysis of the spleen using a monoclonal antibody against Foxp3. As depicted in Figure 7A, the Met-2 regimen significantly reduced the number of regulatory cells presenting a Foxp3 marker. To further investigate the impact of the low level of Tregs in the blood and spleen on other T cells in the tumor microenvironment of Met-2 treated mice, we extracted total RNA from tumors and performed qPCR. Figure 7B shows the mRNA level of CD4^+^, CD8^+^ and FoxP3 T cells in the tumor. In agreement with their level in the blood and spleen of the Met-2 group, CD4^+^ and CD8^+^ lymphocytes were significantly more abundant in the tumor microenvironment in comparison with the saline group. Similarly, the expression of FoxP3, the marker of T regulatory cells, was significantly reduced at the mRNA level in mice treated with the Met-2 schedule (Figure 7B).

### 2.4. The Effect of Low-Dose CTX on Tumor Microenvironment and Its Relationship With TGF-β Signaling Pathway

**Figure 8:**
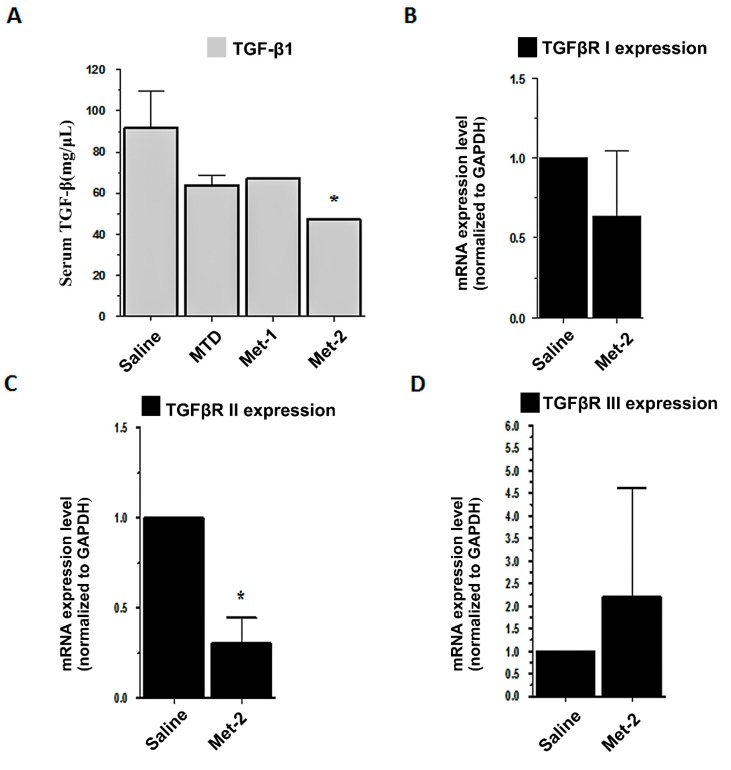
Implication of TGF-β signaling pathway in low cyclophosphamide activity. (**A**) ELISA analysis for serum TGF-β1; (**B**–**D**) qPCR analysis for mRNA expression of TGFβRI, TGFβRII, and TGFβRIII, respectively. Serum was extracted after blood collection and tumors were collected; total RNA was isolated, then qPCR was performed, as previously described, *p < 0.05.

TGF-β is important for Tregs’ maintenance and function. Our results confirm that low-dose CTX can reverse the high number of Tregs not only in circulation but also in the tumor microenvironment. Therefore, we investigated the implication of the TGF-β signaling pathway on the effects of metronomic CTX. First, we performed ELISA analysis of animal serum in order to measure the circulating TGF-β1 level. Figure 8A showed that Met-2 schedules significantly decreased the level of TGF-β1 in the blood. There was no remarkable diminution in the serum level of TGF-β1 in mice treated with either MTD or Met-1 schedules. In addition, qPCR was conducted to evaluate the gene expression of the three TGF-β receptors, including TGFβRI, TGFβRII, and TGFβRIII in the tumor microenvironment. Figure 8B–D demonstrate that the Met-2 schedule significantly reduced only the gene expression of TGFβRII. No change was observed for TGFβRI mRNA in the treated group. The Met-2 regimen increased the level of TGFβRIII mRNA, but not significantly (*p* > 0.05).

It has been widely reported that TGF-β induces a trans-differentiation of fibroblasts and related stromal cells into myofibroblasts, a key play that promotes tumor growth and metastasis [24]. Hence, the effect of metronomic CTX on the tumor microenvironment was further explored by investigating tumor-associated fibroblasts (TAFs), which include both fibroblasts expressing FSP-1 and myofibroblasts with their marker α-SMA, at both the mRNA and protein level.

**Figure 9:**
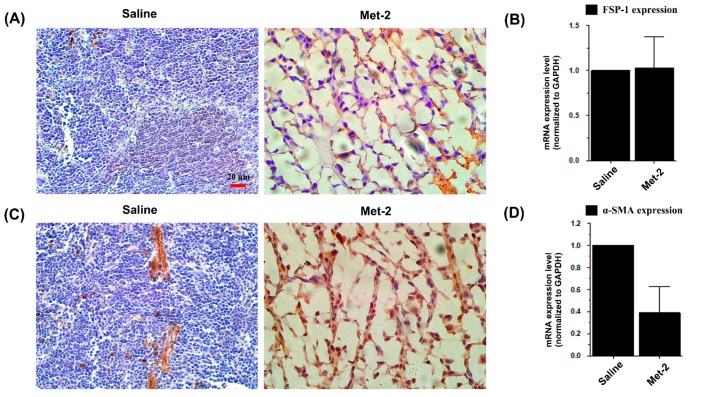
Effect of metronomic cyclophosphamide on tumor-associated fibroblasts (TAFs). (**A**) Immunohistochemistry of FSP-1 in the tumor microenvironment, scale bar = 20 μm. (**B**) qPCR analysis for the mRNA level of FSP-1 in the tumor microenvironment. (**C**) Immunohistochemistry of α-SMA in the tumor microenvironment α-SMA, scale bar = 20 μm. (**D**) qPCR analysis for the mRNA level of α-SMA in the tumor microenvironment. H&E staining was performed as indicated above. Tumors were collected and total RNA was isolated; then, qPCR was performed as previously described.

Immunohistochemical analysis shown in Figure 9 revealed a low level of myofibroblasts and high expression of fibroblast marker in the tumor microenvironment. At the mRNA level, the Met-2 schedule decreased the expression of α-SMA (Figure 9C,D) with no change in FSP-1 expression (Figure 9A,B) in the tumor tissue.

**Figure 10:**
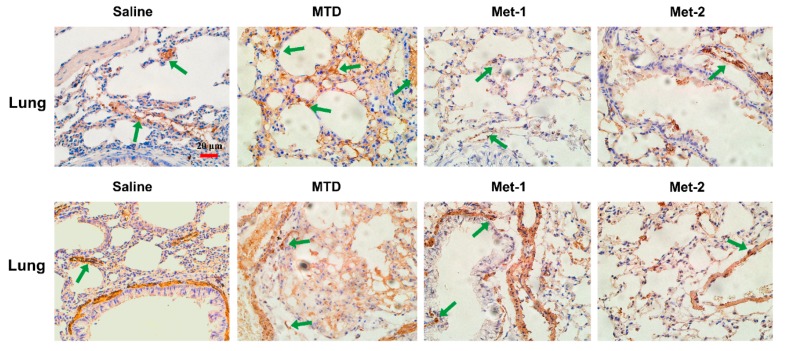
Immunohistochemistry of FSP-1 and α-SMA in the lung. Immunohistochemistry of the lung for FSP-1 marker (showed by green arrow) of fibroblasts and α-SMA marker of myofibroblasts was performed as previously described. Scale bar = 20 μm.

The expression of FSP-1 and α-SMA in the lung, which is a common site for metastases from Lewis Lung Cancer (LLC) cells [25], was also investigated. Recently, the presence of activated myofibroblasts expressing α-SMA, was detected in pulmonary metastases of certain tumors [26]. In our investigation, Met-2 treated mice had less α-SMA expression in the lung, in comparison to the saline group (Figure 10). Meanwhile, there was no significant increase in the FSP-1 marker in lungs of Met-2 mice (Figure 10). On the contrary, lungs from animals treated with either MTD or Met-1 regimens had a significant expansion of α-SMA expression, in comparison to both saline and Met-2 groups. Interestingly, Met-1 tissues had a very low FSP-1 expression (Figure 10).

**Figure 11:**
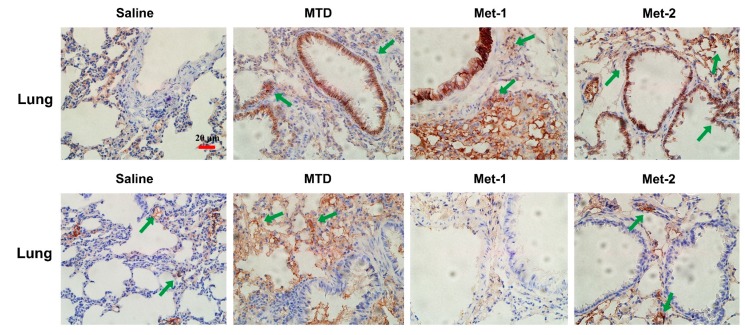
Immunohistochemistry of E-cadherin and N-cadherin in the lung(showed by green arrow). Immunohistochemistry of the lung for E-cadherin and N-cadherin markers of EMT was performed as previously described. Scale bar = 20 μm.

Because the Met-2 regimen decreased the expression of α-SMA in both primary tumor site and lung, we feel it may inhibit epithelial to mesenchymal transition (EMT), a phenomenon that contributes to increased tumor cell motility and invasive behavior. EMT is generally characterized by reduced E-cadherin and increased N-cadherin expression [27]. Figure 8 demonstrates that both metronomic schedules Met-1 and Met-2 increased the level of E-cadherin and mitigated that of N-cadherin. In contrast, the MTD regimen did not display any significant change, in comparison to the saline group (Figure 11).

## 3. Discussion

Conventional therapy protocols are usually based on the “maximum tolerated dose” (MTD) approach, which always requires rest periods between cycles of therapy so that normal hematopoietic cells can survive toxic effects of the drug. However, during this inter-therapy period, some endothelial cell progenitors (ECPs) migrate to the previous tumor site and favor the rebirth of new tumors from resistant clones [28]. In order to avoid such issues and minimize the high toxicity caused by conventional chemotherapeutic schedules, a new concept of drug administration called “metronomic chemotherapy” or “low dose” regimen was introduced [29,30]. Metronomic chemotherapy refers to the frequent administration of chemotherapeutic agents at low dosage [31]. The benefit of this therapy lies not only in its anticancer efficacy and low toxicity, but also in cellular target switch toward endothelial cells, as proposed by Folkman and Kerbel [21,32].

Growing evidence indicates that chemotherapeutic drugs, such as cyclophosphamide, when giving at low doses, display important immunostimulatory properties by targeting regulatory T cells (Tregs), a major player favoring tumor progression and therapy resistance [32]. Data from both animal and clinical studies have shown that low-dose cyclophosphamide (CTX) can ameliorate anti-tumor immune responses by reducing both the frequency of circulating Tregs and their immunosuppressive functions, therefore increasing both lymphocyte proliferation and memory T cells [21,22,23].

In our study, one high-dose and two low-dose regimens of CTX were given to mice harboring LLC cells. We initially assessed the toxicity of different regimens; we found that the Met-2 schedule, which consists of continuous low-dose treatment with CTX, had the least toxic effect, as reflected by the high survival rate, low body-weight loss, and absence of tissue damage in multiple organs. Another metronomic schedule Met-1, which was first described by T. Browder et al., is based on administration of the MTD at longer intervals than those of conventional therapy. Although this regimen extended animal survival—as reported previously [9]—with no remarkable body weight change, it showed some toxicity, mainly in the heart. As anticipated, MTD-treated mice had more body weight loss, low survival, and evident toxicity in multiple tissues.

Further, the antitumor activity of the three therapeutic doses was explored. The MTD and Met-1 schedules had the most striking antitumor effect, as shown in Figure 3. The Met-2 schedule’s antitumor effect was also strong, but less than that of MTD and Met-1.

Because low-dose CTX can boost the host immune system function by targeting regulatory T cells (Tregs) [21,22,23], we evaluated the percentages of CD4^+^ T cells, CD8^+^ T cells, and Tregs after treatment with CTX. Our data demonstrates that low and continuous treatment with CTX could restore the loss of CD4^+^, CD8^+^ T populations at both the gene and protein levels. Although an early study conducted by Motoyoshi et al. indicated that low dose administration of CTX to tumor-free C3H/HeN mice resulted in a notable decrease of these cells [33], it is worth mentioning that in clinical settings, metronomic cyclophosphamide treatment for three months caused a short-term Tregs reduction in the peripheral blood of breast cancer patients, along with an enhanced expansion of CD4^+^ and CD8^+^ T effector cells [34].

Additionally, we found that the Met-2 schedule reduced the number of Tregs not only in the circulatory and splenic compartment, but also in the tumor microenvironment. This is in agreement with observations demonstrating depletion of circulating Treg population after low CTX therapy [22,23].

On the other side, the Met-1 and MTD regimens caused a significant reduction of both CD4^+^ and CD8^+^ populations. Interestingly, the loss of both lymphocytic populations was accompanied by a high frequency of regulatory T cells. Further investigation is necessary to explain this finding.

Transformation growth factor beta (TGF-β) plays a critical role in maintaining, activating, and supporting Tregs in their function [35,36]. Because metronomic CTX specifically targets this population of cells, we hypothesized that this effect may be due to the modulation of TGF-β. Studies have demonstrated that the administration of cyclophosphamide to tumor-bearing animals reduced the production of TGF-β [8,15]. Our results were consistent with these observations. Interestingly, besides lowering circulating TGF-β, the Met-2 treatment was able to decrease the gene expression of TGFβRII significantly, as revealed by the qPCR results. This is the first time low dose cyclophosphamide is shown to modulate TGF-β pathway by downregulating one of its receptors, which may hold promise for further research on the mechanism of action of metronomic chemotherapy at the molecular level. The actions of TGF-β in the tumor microenvironment are diverse. For instance, TGF-β controls myofibroblasts induction from fibroblasts [6] and from transdifferentiation by epithelial-to-mesenchymal transition (EMT) [7]. These myofibroblasts are usually detected in pulmonary metastases of certain tumors [26]. Here, we showed that low and continuous administration CTX could reduce the number of these cells not only in the primary tumor but also in the lung. Finally, metronomic CTX increased the level of E-cadherin and mitigated that of N-cadherin, which may, to some extent, indicate the implication of the TGF-β pathway.

In conclusion, only low and continuous administration of cyclophosphamide could reduce tumor growth with low toxicity. This effect was mainly due to restoration of immune function via increase of CD4^+^/CD8^+^ T cells and depletion of regulatory T cells (Tregs). The depletion of Tregs in both blood and primary tumors was accompanied by a reduction of myofibroblasts and EMT markers in the tumor and lung, which may be explained by the low level of TGF-β and downregulation of TGF-β receptor 2 (TGFβRII).

## 4. Materials and Methods

### 4.1. Reagents

Cyclophosphamide (CTX) was purchased from ZhongDa Hospital (Nanjing, CHINA). The drug was dissolved in sterile saline and given intraperitonealy (i.p) following 3 schedules: MTD regimen (150 mg/kg, 3 doses the first week, followed by 2 weeks’ break), metronomic schedule 1 (Met-1, 170 mg/kg every 6 days for 2 cycles) [9], and metronomic schedule 2 (Met-2, 25 mg/kg every other day) [37].

RPMI-1640 (Life Technologies, Inc., Grand Island, USA); fetal bovine serum (Hyclone, Australia); Trypsin (Solarbio, Beijing, China), Trypan blue (Invitrogen, Carlsbad, California, USA), Human/Mouse TGF-β1 ELISA kit (eBioscience, San Diego, USA); The PrimeScript RT Master Mix (Perfect Real Time) and SYBR Premix Ex Taq II (Tli RNaseH Plus) kits (TaKaRa, Dalian, China); TRIzol (Invitrogen, Carlsbad, California, USA); Primers for GAPDH, CD4, CD8, FoxP3, α-SMA, FSP-1, TGFβRI, TGFβRII and TGFβRIII (Wei Wo Biotech, Nanjing, China).

### 4.2. Cell Culture

Lewis lung cancer (LLC) cells were kindly provided by Guo QingLong from China Pharmaceutical University. The cells were maintained in RPMI-1640 medium (Life Technologies, Inc., Grand Island, USA) supplemented with 10% (*v*/*v*) heat-inactivated fetal bovine serum, 2 mm glutamine, 10 mM HEPES buffer (4-(2-hydroxyethyl)-1-piperazineethanesulfonic acid), 100 U/mL streptomycin, and 100 U/mL penicillin at 37 °C in a humid atmosphere (5% CO_2_, 95% air). For the subculture, cells were detached with 0.25% trypsin-EDTA (Ethylenediamine tetra-acetic acid) solution, and 1 × 10^6^ cells were seeded into new flasks.

### 4.3. Mouse LLC Lung Cancer Model

Male C57BL/6J mice were purchased at 6 to 8 weeks of age from Nanjing JunKe Biotech Animal laboratory (Nanjing, China). The animals were housed in a room with constant temperature (23 °C) and 12-h light/dark cycle with free access to standard mouse chow and water. They were kept in sterile conditions for 10 days of environment adaptation. On the day of the injection, tumor cells were harvested and cell viability was determined by trypan blue dye exclusion on a Countess™ automated cell counter (Life Technologies, Invitrogen, Grand Island, CA, USA); it was greater than 95%. The mice were injected subcutaneously with 1 × 10^6^ LLC cells in 100 μL saline in the right flank. Ten days later, tumor cells from the first generation of tumor-bearing mice were inoculated in new animals for another 10 day period. Finally, the second generation mice were sacrificed and cells were injected in the study group. Six days later, when the tumors were palpable (tumor volume ≈ 100 mm^3^), the mice were randomly divided into 4 groups (n = 10 animals/group): Saline group, MTD group, metronomic 1 (Met-1) group, and metronomic 2 group (Met-2), following the schedules mentioned above (Materials and Methods section). Tumor width (W) and length (L) were measured every other day by calipers. Tumor volume was calculated according to the following formula: Tumor volume = 0.52 × L × W^2^, where L is the length and W is the width of the tumors. The mice in all groups were sacrificed 21 days after treatment. All experiments were done according to the Guides for the Care and Use of Laboratory Animals and approved by China Pharmaceutical University Animal Care Committee.

### 4.4. Flow Cytomerty Analysis

Flow cytometry analysis for circulating CD4^+^, CD8^+^ and CD4^+^CD25^+^FoxP3 was performed on both fresh blood and spleen using a kit (eBioscience, San Diego, USA) following the manufacturer’s protocol. Briefly, just after the animals were sacrificed, fresh blood was collected in small tubes filled with 1/10 *v*/*v* EDTA-2Na to stop coagulation. For CD4^+^, CD8^+^ T cells, to a 100 µL of each sample, 1 µL of anti-mouse CD4-FITC and 1 µL of anti-mouse CD8-PE was added and the mixtures were kept in dark at 4 °C for 20 min. Lysis buffer was then added and the samples were further incubated in dark at 4 °C for 10 min. The mixtures were centrifuged at 1500 rpm for 3–5min and washed with PBS once. Finally, 200 µL of PBS was added to each sample and FACS analysis was carried out using a FACSCalibur flow cytometer. For CD4^+^CD25^+^FoxP3 T cells, 1 µL of anti-mouse CD4-FITC and 1 µL of anti-mouse CD25-PE were added and the mixtures were kept in dark at 4 °C for 30 min. After washing cells in cold flow cytometry staining buffer, the peller was centrifuged and the supernatant was discarded. Resuspended cells in freshly prepared fixation/permeabilization working solution were again incubated at 4 °C for between 30 min and 18 h in the dark. Another series of washing was performed and cells were stained with anti-mouse Foxp3-PE-Cy5 (FJK-16s) antibody at 4 °C for at least 30 min in the dark. The samples were washed again and resuspended in an appropriate volume of flow cytometry staining buffer and analyzed on a flow cytometer.

To analyze the spleen’s lymphocytes, tissues were placed in a small beaker containing 1–2 mL of PBS and cut extensively until a homogeneous liquid was formed, which was treated as fresh blood.

### 4.5. Histopathological Analysis

Hematoxylin and eosin (H&E) staining was performed on the heart, liver, lung, kidney, spleen, thymus, and tumor. Tissues were collected after sacrificing the animals and were fixed in 10% paraformaldehyde solution. Sections of 3–5 μm were made from the paraffin-embedded tissues and staining was performed in accordance with standard procedures at Jiangsu Provincial Integrated Chinese-Western Medicine Hospital.

### 4.6. Immunohistochemistry

Sections of 5 μm thickness paraffin blocks were placed on adhesive-coated slides. Then, the sections were deparaffinized with xylene, rehydrated in graded ethanol (from 100% to 70%), and heated for 30 min in a sodium citrate buffer to increase epitope exposure. Additionally, the slides were treated with 0.3% (*v*/*v*) H_2_O_2_ in for 5 min, washed with 0.01 M PBS, and blocked with 1% BSA, 0.2% Tween 20 in PBS for 1 h at room temperature. The slides were then incubated overnight at 4 °C with monoclonal antibody against: Foxp3 (Cell Signaling, Danvers, MA), monoclonal antibody against fibroblast surface protein 1, FSP-1 (Abcam, Cambridge, UK), monoclonal antibody against alpha smooth muscle actin, α-SMA (Abcam, Cambridge, UK), and monoclonal antibodies against E-cadherin and N-cadherin (Cell Signaling, Danvers, MA). The optimal dilution for all antibodies was 1:100. The reaction antigen-antibody was visualized with avidin-biotin peroxidase by using 3,3-diaminobenzidine as the chromogen, which resulted in brown staining. The slides were counterstained in Harris hematoxylin. The samples were then dehydrated, preserved with a cover slip, and reviewed using light microscopy. Pictures were viewed using Olympus FLUOVIEW Viewer software (Tokyo, Japan).

### 4.7. qPCR Analysis

Total mRNA from the animal tissues was isolated using TRIzol Reagent (Invitrogen, Carlsbad, California, USA). RNA was reverse-transcribed using The PrimeScript RT Master Mix (Perfect Real Time) (TaKaRa, Dalian, China). Real-time quantitative RT-PCR was performed using the iCycler 5 thermal cycler (BioRad, California, USA). Each cDNA was amplified in a 20 µL volume using the SYBR Premix EX Taq kit (TaKaRa, Dalian, China), with a 500 nM final concentration of each primer. The amplification specificity was checked using melting curve analysis. For each cDNA, all target gene mRNA levels were normalized to GAPDH mRNA levels. Results are expressed as the ratio of normalized target gene mRNA levels in the treated groups relative to those in the saline group. For real-time PCR, the following primer sets were used:
Mouse GAPDH, (NCBI RefSeq: NM_008084.2):  Forward: 5’-CTTTGGCATTGTGGAAGGGCTC-3’  Reverse: 5’-GGCATGGACTGTGGTCATGA-3’Mouse CD4, (NCBI RefSeq: NM_008084.2):  Forward: 5’-GAGAACAGGAAAGAGGAGGTGGAGT-3’  Reverse: 5’-GGAGAGAACTTTGGAACCACTGACA-3’Mouse CD8, (NCBI RefSeq: NM_008084.2):  Forward: 5’-AAGCCCAGACCTTCAGAGAAAATT-3’  Reverse: 5’-CCCATCACACCCCTACTAAAACAA-3’Mouse Foxp3, (NCBI RefSeq: NM_001199347.1):  Forward: 5’-ACCTATGCCACCCTTATCCGA-3’  Reverse: 5’-CGAACATGCGAGTAAACCAATG-3’Mouse alpha smooth muscle actin (α-SMA), (NCBI RefSeq: NM_007392.3):  Forward: 5’-CCACTGAACCCTAAGGCCAAC-3’  Reverse: 5’-CTCCAGAGTCCAGCACAATACCA-3’Mouse fibroblast specific protein (FSP-1) (NCBI RefSeq: NM_011311.2):  Forward: 5’-GTGTCCACCTTCCACAAATACTCAG-3’  Reverse: 5’-AATGCAGCTTCATCTGTCCTTTTC-3’Mouse transforming growth factor, beta receptor I (Tgfbr1) (NCBI RefSeq: NM_009370.2):  Forward: 5’-CTCTGTTTTTCCCACTCTGCC-3’  Reverse: 5’-GCTTCCATCAATCACTTCATTTTAG-3’Mouse transforming growth factor, beta receptor II (Tgfbr2) (NCBI RefSeq: NM_009371.3):  Forward: 5’-TCCAGACTTCCCATTACTCACACC-3’  Reverse: 5’-TCTCCATGCTCACGAACGCAC-3’Mouse transforming growth factor, beta receptor III (Tgfbr3) (NCBI RefSeq: NM_011578.3):  Forward: 5’-GTCCCTGTGTTTGTCCTGATGAG-3’  Reverse: 5’-CCAGACAGAACGGTGAAGCTCTC-3’

### 4.8. Mouse TGF-β1 ELISA Analysis

The concentrations of TGF-β1 in the serum were determined by ELISA kits (eBioscience, San Diego, USA) according to the manufacturer’s protocols. Briefly, serum samples were diluted 1/5 in phosphate buffer saline (PBS) and acidified by 1 N solution of HCL in order to activate latent TGF-β1 to the immunoreactive form. The samples were then neutralized using 1 N solution of NaOH. Activated samples and standard curve dilutions were added into a 96 well plate and allowed to react with the capture antibody overnight at 4 °C. After a series of aspiration/washing, a diluted detection antibody was added and the plate incubated at room temperature for 1 h. The wells were washed again and the enzyme Avidin-HRP was added. After incubation at room temperature for 30 min, the plate was washed and a substrate solution introduced into each well. The reaction was stopped 15 min later using a stop solution (2 N H_2_SO_4_) and absorbance was read as 450 nm. Samples concentrations were determined using an equation obtained from the standard curve. Data were represented as Mean± S.D. (standard of deviation).

### 4.9. Statistical Analysis

Data are represented as Mean ± S.D. (standard of deviation). The *t*-test was used for statistical analysis between the two groups. Significant differences were accepted when *p* < 0.05. A *p* value ≤ 0.05 was considered significant (*or # *p* ≤ 0.05, ** or ## *p* ≤ 0.01, *** or ### *p* ≤ 0.001).
